# Overweight and obesity significantly increase colorectal cancer risk: a meta-analysis of 66 studies revealing a 25–57% elevation in risk

**DOI:** 10.1007/s11357-024-01375-x

**Published:** 2024-10-08

**Authors:** Zoltan Ungvari, Mónika Fekete, Peter Varga, Andrea Lehoczki, János Tibor Fekete, Anna Ungvari, Balázs Győrffy

**Affiliations:** 1 Vascular Cognitive Impairment, Neurodegeneration and Healthy Brain Aging Program, Department of Neurosurgery, University of Oklahoma Health Sciences Center, Oklahoma City, OK USA; 2 Stephenson Cancer Center, University of Oklahoma, Oklahoma City, OK USA; 3 Oklahoma Center for Geroscience and Healthy Brain Aging, University of Oklahoma Health Sciences Center, Oklahoma City, OK USA; 4 Department of Health Promotion Sciences, College of Public Health, University of Oklahoma Health Sciences Center, Oklahoma City, OK USA; 5 International Training Program in Geroscience, Doctoral College/Institute of Preventive Medicine and Public Health, Semmelweis University, Budapest, Hungary; 6 Institute of Preventive Medicine and Public Health, Semmelweis University, Semmelweis University, Budapest, Hungary; 7 Dept. of Bioinformatics, Semmelweis University, 1094 Budapest, Hungary; 8 Cancer Biomarker Research Group, Institute of Molecular Life Sciences, HUN-REN Research Centre for Natural Sciences, 1117 Budapest, Hungary; 9 Dept. of Biophysics, Medical School, University of Pecs, 7624 Pecs, Hungary

**Keywords:** Epidemiology, Aging, Age-related disease, Malignancy, Neoplasm, Adiposity, Adipose, Colon carcinoma

## Abstract

The incidence of colorectal cancer (CRC) has been steadily rising, and obesity has been identified as a significant risk factor. Numerous studies suggest a strong correlation between excess body weight and increased risk of CRC, but comprehensive quantification through pooled analysis remains limited. This study aims to systematically review and meta-analyze the existing literature to evaluate the association between obesity and CRC risk, considering variations across sex and study designs. A systematic literature search was conducted in PubMed, Cochrane Central Register of Controlled Trials (CENTRAL), and Web of Science to identify randomized controlled trials and human clinical trials from 1992 to 2024. Statistical analysis was performed using the https://metaanalysisonline.com web application using a random effects model to estimate the pooled hazard rates (HR). Forest plots, funnel plots, and Z-score plots were utilized to visualize results. We identified 52 clinical trials and 14 case–control studies, encompassing a total of 83,251,050 and 236,877 subjects, respectively. The pooled analysis indicated that obesity significantly increased the prevalence of CRC (HR = 1.36, 95% CI = 1.24–1.48, *p* < 0.01). This effect was consistent across sexes, with HRs of 1.57 (95% CI = 1.38–1.78, *p* = 0.01) for males and 1.25 (95% CI = 1.14–1.38, *p* < 0.01) for females. Case–control studies specifically showed an effect, but with marginal significance only (HR = 1.27, 95% CI = 0.98–1.65, *p* = 0.07). The Z-score plot indicated the need for additional analysis in the case–control group. A significant heterogeneity was observed across studies in all four settings. This meta-analysis provides robust evidence that obesity is a significant risk factor for colorectal cancer, with an overall hazard rate indicating a 36% increased risk. The effect is pronounced across both sexes, with males showing a slightly higher risk compared to females. Although case–control studies showed a weaker association, the overall trend supports the link between obesity and CRC. These results underscore the importance of public health interventions aimed at reducing obesity to potentially lower the risk of colorectal cancer.

## Introduction

Colorectal cancer (CRC) remains one of the leading causes of cancer-related morbidity and mortality globally, with particularly high incidence rates in the European Union (EU) [1–5]. CRC is predominantly an age-related disease, with most cases occurring in individuals over the age of 50 [2, 4, 6]. This trend underscores the significant role of fundamental cellular and molecular aging processes in the development of CRC [6, 7].

Numerous studies have identified a variety of risk factors for CRC [8, 9], among which overweight and obesity are particularly prominent [10–75]. The growing prevalence of overweight and obesity has raised significant public health concerns [76], necessitating a deeper understanding of their impact on CRC risk. The obesity epidemic is a global phenomenon, with particularly high prevalence rates observed in developed regions such as the United States and the European Union (EU) [77, 78]. In the United States, the prevalence of obesity among adults has more than doubled since the 1970s, with current estimates indicating that over 40% of adults are obese [79–84]. Similarly, the EU faces a substantial obesity burden, with significant variations in obesity rates among member states [8, 78]. In 2019, the proportion of overweight adults in the EU varied significantly: for women, it ranged from 37% in Italy to 58% in Croatia, and for men, it ranged from 53% in France to 73% in Croatia [78]. Notably, Hungary stands out as one of the most obese nations in the EU, with nearly two-thirds of its adult population classified as overweight or obese in 2019 (67.3% for males and 53.3% for females) [78]. The prevalence of overweight and obesity among older adults is even more alarming: over 68% of adults aged 45 to 64, 76.4% of adults aged 65 to 74, and 67.3% of adults aged 75 and older were overweight or obese in 2019 [78]. Comparable trends are evident in other EU countries and the United States [84, 85], where high levels of overweight and obesity among both adults and the elderly signify a broader public health concern.

As the population ages, the intersection of obesity and aging [79] becomes increasingly critical in understanding CRC risk. Older adults are particularly vulnerable to obesity-related health issues [86–96], including CRC, as the cumulative effects of prolonged obesity can exacerbate age-related cellular damage, senescence, and inflammation [97–103]. Accordingly, there is emerging data suggesting that obesity in aging populations may pose a more significant risk for CRC compared to younger individuals. Epidemiological data show that the incidence rates of CRC rise sharply with age. When stratified by BMI, these rates are significantly higher in obese elderly populations compared to their normal-weight counterparts [5]. This alarming trend underscores the urgency of addressing obesity as a major public health issue. The implications are particularly profound for countries like the United States and Hungary, where the high prevalence of obesity could have significant repercussions for CRC incidence and outcomes. Understanding the link between overweight, obesity, and CRC risk is crucial for developing effective prevention and intervention strategies.

This meta-analysis aims to provide a comprehensive evaluation of the association between overweight and obesity and the risk of colorectal cancer. By analyzing data from 66 studies, we seek to quantify the increased CRC risk associated with different levels of excess body weight. Our findings are expected to provide critical insights that will inform public health policies and interventions, particularly in regions with high obesity rates, to help mitigate the growing burden of colorectal cancer.

## Methods

### Search strategy

**Fig. 1:**
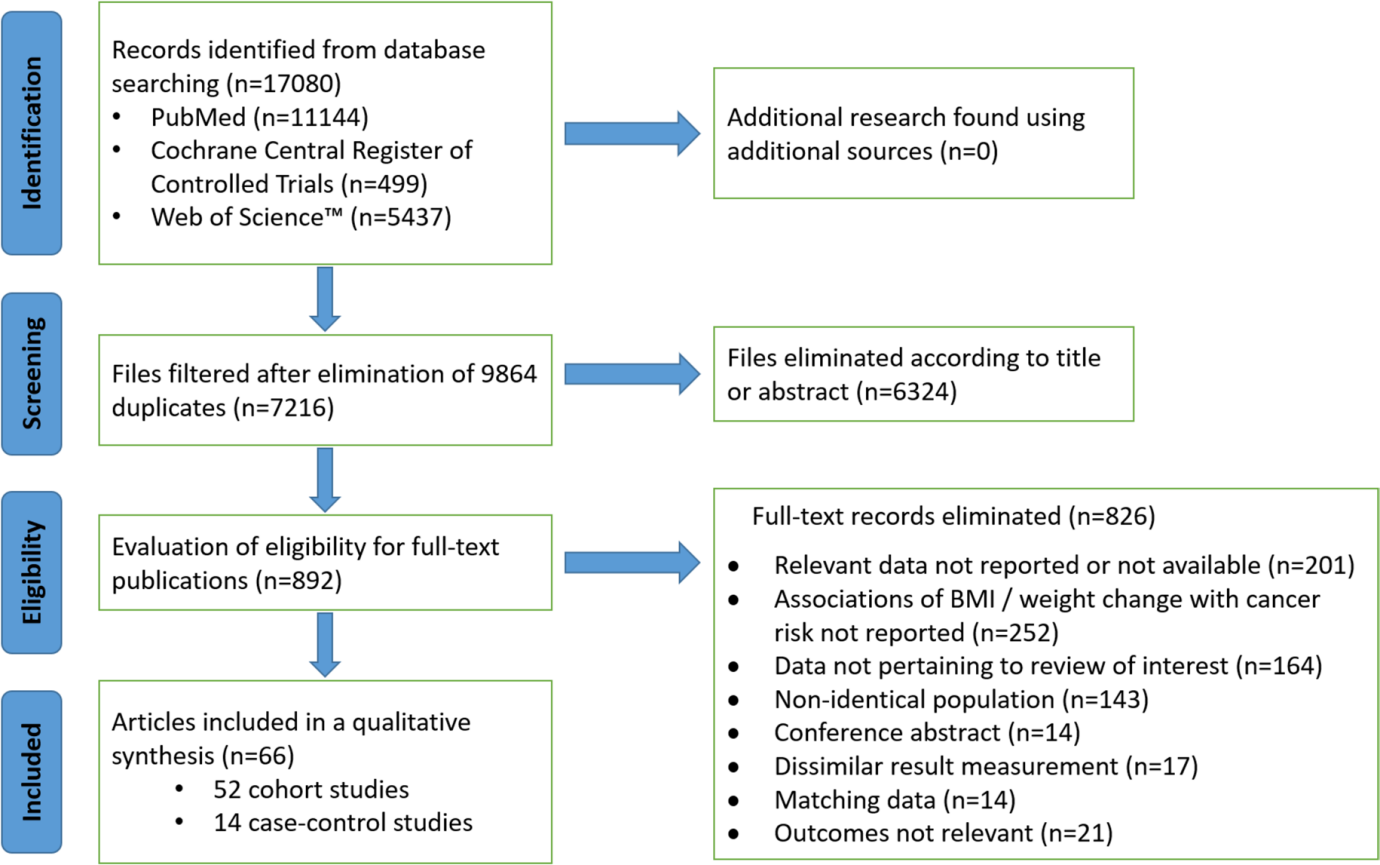
Flow diagram of article selection process

We conducted a systematic search of the PubMed, Cochrane Central Register of Controlled Trials (CENTRAL), and Web of Science databases from 1992 to 2024 to identify studies examining the associations between overweight and obesity and the risk of colorectal cancer [10–61] (Fig. 1). The search terms used included “colorectal cancer,” “body mass index,” overweight, and obesity. Table 1 contains the combination of search terms used for the systematic review of overweight, obesity, and colorectal cancer risk. We excluded studies on cancer precursors such as colorectal adenomas because our primary objective was to evaluate the risk of CRC than precursor lesions. While adenomas are a known risk factor for CRC, including them could introduce variability due to the different natural histories and progression rates of adenomas to CRC.

**Table 1:** Search terms for systematic review on overweight, obesity, and colorectal cancer risk

Search focus	Search terms
Colorectal cancer and body mass index (BMI) in cohort studies	“colorectal cancer AND body mass index AND cohort study”
Colorectal cancer and obesity in case–control studies	“colorectal cancer AND obesity AND case–control study”
BMI and colorectal cancer risk in meta-analyses	“BMI AND colorectal cancer risk AND meta-analysis”
Obesity and colorectal cancer incidence in systematic reviews	“obesity AND colorectal cancer incidence AND systematic review”
Overweight and colorectal cancer risk estimates	“overweight AND colorectal cancer risk estimates”
BMI, obesity, and CRC risk	“BMI AND obesity AND colorectal cancer risk”
Body mass index and CRC incidence	“body mass index AND colorectal cancer incidence”
Colorectal cancer and adiposity	“colorectal cancer AND adiposity”
Risk estimates for CRC related to obesity	“risk estimates AND colorectal cancer AND obesity”
Hazard ratios for BMI and CRC	“hazard ratios AND body mass index AND colorectal cancer”
Relative risk of CRC with obesity	“relative risk AND colorectal cancer AND obesity”

### Study eligibility assessment

The eligibility of each study was independently assessed by two researchers (AU, MF) [10–75]. We excluded studies that were not published as full reports, such as conference abstracts and letters to editors, studies focusing on cancer mortality (rather than incidence), and studies of cancer precursors (e.g., colorectal adenoma and/or polyps). The inclusion criteria for the studies incorporated into the meta-analysis are outlined in Table 2.

**Table 2:** Inclusion criteria for studies included in the meta-analysis

Criteria	Description
Cohort studies	Included cohort studies that determined BMI at baseline and recorded cancer cases during follow-up
Risk estimates	Required each cohort study to report risk estimates (relative risks, odds ratios, or hazard ratios) with 95% confidence intervals, separately for men, women, or both
Case–control studies	Included nested case–control studies within cohort studies
Height and weight data	Included studies where height and weight (for BMI calculation) were self-reported or directly measured

### Determining the overall effect

Statistical analysis was conducted using the web application available at https://metaanalysisonline.com. The random effects model was utilized to estimate pooled hazard rates (HR), odds ratios (OR), and their 95% confidence intervals (CI). Forest plots were generated to visualize both individual studies and summary results, providing a graphical representation of data variability and the overall effect estimate. Heterogeneity among the included studies was evaluated using the chi-squared test and I^2^ index.

Funnel plots were created to assess the relationship between the estimated effects from each study and their precision, and to examine publication bias. Egger’s test was performed to determine the significance of this bias.

### Determining sample size robustness

Trial sequential analysis (TSA) was conducted to evaluate the robustness of the sample size. The a priori information size (APIS) was determined under a 10% risk ratio reduction with a two-sided α of 5% and a power (1 – β) of 80%. TSA analyses were performed in Stata 14.1 using the metacoumbounds package. A Z-score plot was created to visualize the relationship between the cumulative sample size, time, and cumulative Z-scores. This analysis helped assess whether the cumulative sample size was sufficient for conclusive inference or if additional studies were necessary.

### Subcohort analysis settings

To provide a comprehensive understanding, we conducted the statistical analysis across several specific settings. First, we performed a combined analysis of all included studies to generate an overall effect estimate. Next, we carried out two separate analyses for men and women to explore potential gender-specific differences in the outcomes. Finally, we analyzed case–control studies independently, where individuals with colorectal cancer (cases) were compared to those without (controls). This multifaceted approach allowed us to assess the robustness and applicability of our findings across different subgroups and study designs.

## Results

### Cohort studies for colorectal cancer (both sexes)

A total of 32 studies were analyzed incorporating results from both sexes [11, 18, 22, 27, 28, 32, 33, 35, 37–54, 56–61]. Using the random effects model with the inverse variance method to compare the hazard rates (HR), a statistically significant difference was found, with a summarized hazard rate of 1.36 and a 95% confidence interval of 1.24–1.48. The test for overall effect indicated significance at *p* < 0.05.

**Fig. 2:**
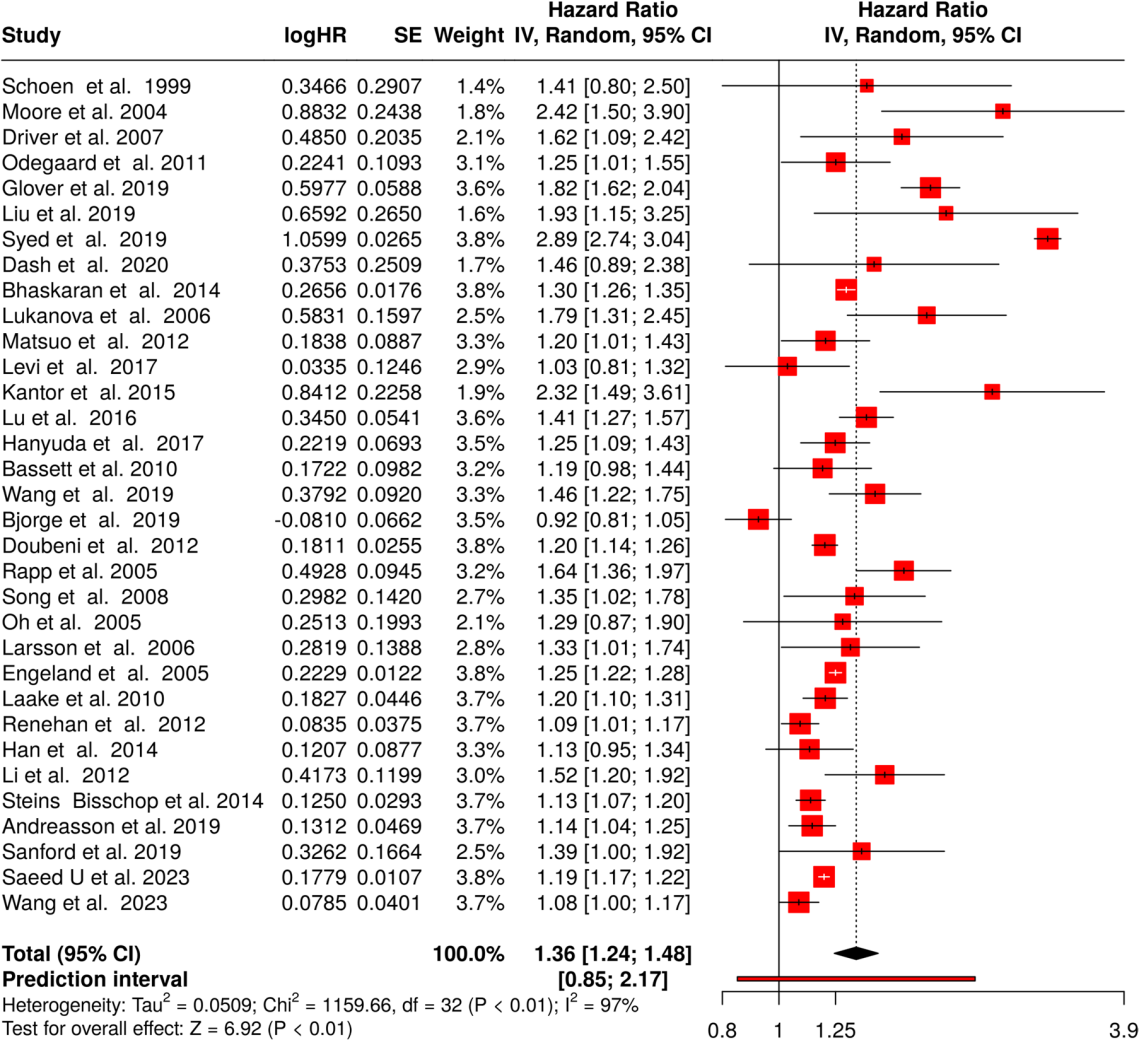
Meta-analysis of cohort studies linking obesity and colorectal cancer in both sexes published between 1999 and 2023 shows a highly significant effect. HR, hazard rate; SE, standard error; CI, confidence interval; IV, inverse variance

Significant heterogeneity was detected, suggesting inconsistent effects in magnitude and/or direction among the studies. The I^2^ value of 97.2% indicates that most of the variability among studies is due to heterogeneity rather than random chance (see Fig. 2).

**Fig. 3:**
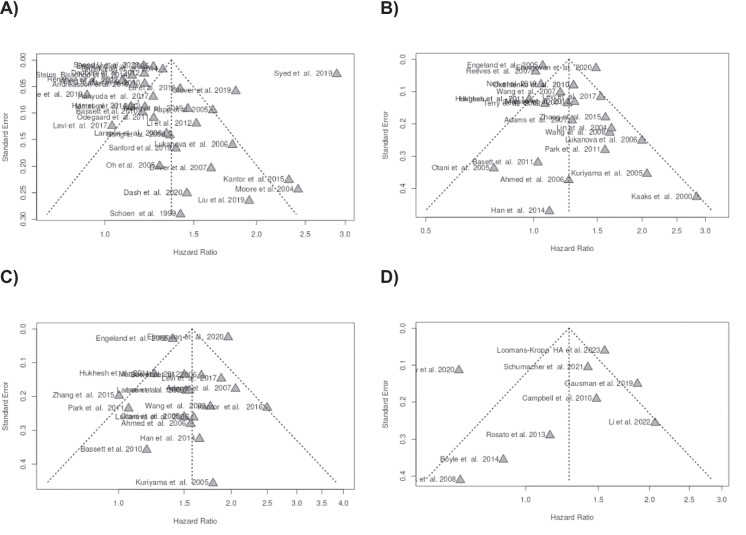
Funnel plots indicate no potential publication bias in the four different setting analyzed, including cohort studies for CRC in both sexes (**A**), cohort studies for CRC in women (**B**), cohort studies for CRC in man (**C**), and case–control studies for CRC in both sexes (**D**)

The funnel plot does not suggest potential publication bias. Egger’s test does not support the presence of funnel plot asymmetry (intercept: 1.04, 95% CI − 1.76–3.84, t: 0.727, *p*-value: 0.473, depicted in Fig. 3A).

### Cohort studies for colorectal cancer—women

A total of 23 trials were included in the analysis [10–32]. Using the random effects model with the inverse variance method to compare hazard rates, a significant change was found, with a summarized hazard rate of 1.25 and a 95% confidence interval of 1.14–1.38. The test for overall effect showed significance at *p* < 0.05.

**Fig. 4:**
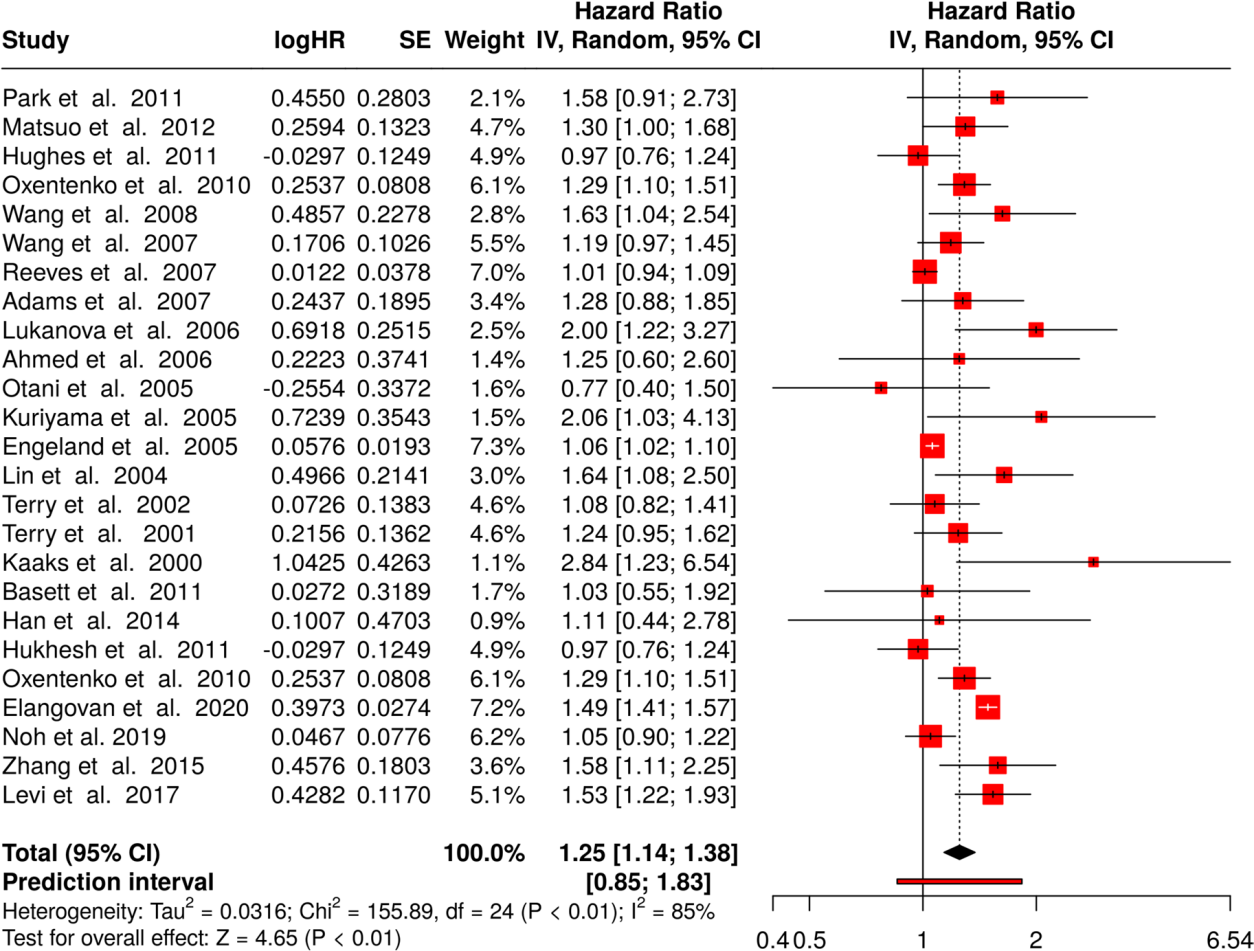
Meta-analysis of cohort studies linking obesity and colorectal cancer in women published between 2001 and 2023 shows a significant effect. HR, hazard rate; SE, standard error; CI, confidence interval; IV, inverse variance

We observed a noteworthy heterogeneity, suggesting varying effects in scale and/or direction between the trials. The I^2^ value of 84.6% specifies that most of the variability among studies is due to heterogeneity rather than accidental chance (presented in Fig. 4).

Based on the funnel plot, there is no publication bias. Egger’s test does not support the presence of funnel plot asymmetry (intercept: 0.78, 95% CI − 0.54–2.1, t: 1.159, *p*-value: 0.258; shown in Fig. 3B).

### Cohort studies for colorectal cancer—men

A total of 20 studies were used in this breakdown [10–12, 14, 17–22, 27–29, 31–36, 55]. Using the random effects model with the inverse variance method to liken hazard rates, a statistically significant difference was uncovered, with a summarized hazard rate of 1.57 and a 95% confidence interval of 1.38–1.78. The test for overall effect indicated significance at *p* < 0.05.

**Fig. 5:**
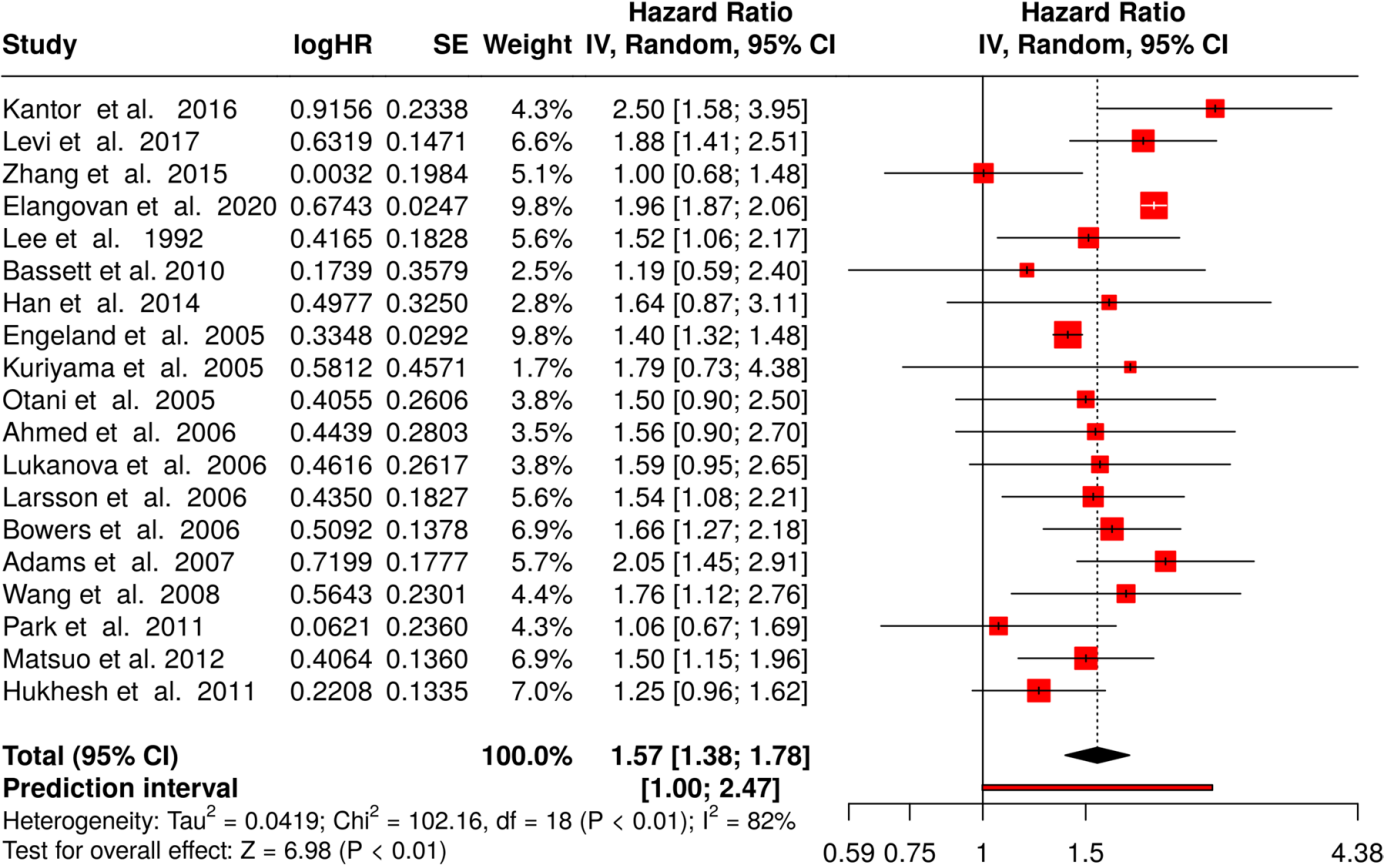
Meta-analysis of cohort studies linking obesity and colorectal cancer in man published between 1992 and 2023 shows a highly significant effect. HR, hazard rate; SE, standard error; CI, confidence interval; IV, inverse variance

Notably, a substantial heterogeneity was present, suggesting varying effects in magnitude and/or direction amongst the studies. The I^2^ value of 82.4% indicates that most of the variability among the results is due to heterogeneity rather than casual chance (see Fig. 5).

The funnel plot does not suggest a likely publication bias. Egger’s test does not back a significant funnel plot asymmetry (intercept: − 0.6, 95% CI − 1.99–0.79, t: − 0.843, *p*-value: 0.411; displayed in Fig. 3C).

### Case–control studies for colorectal cancer—both sexes

**Fig. 6:**
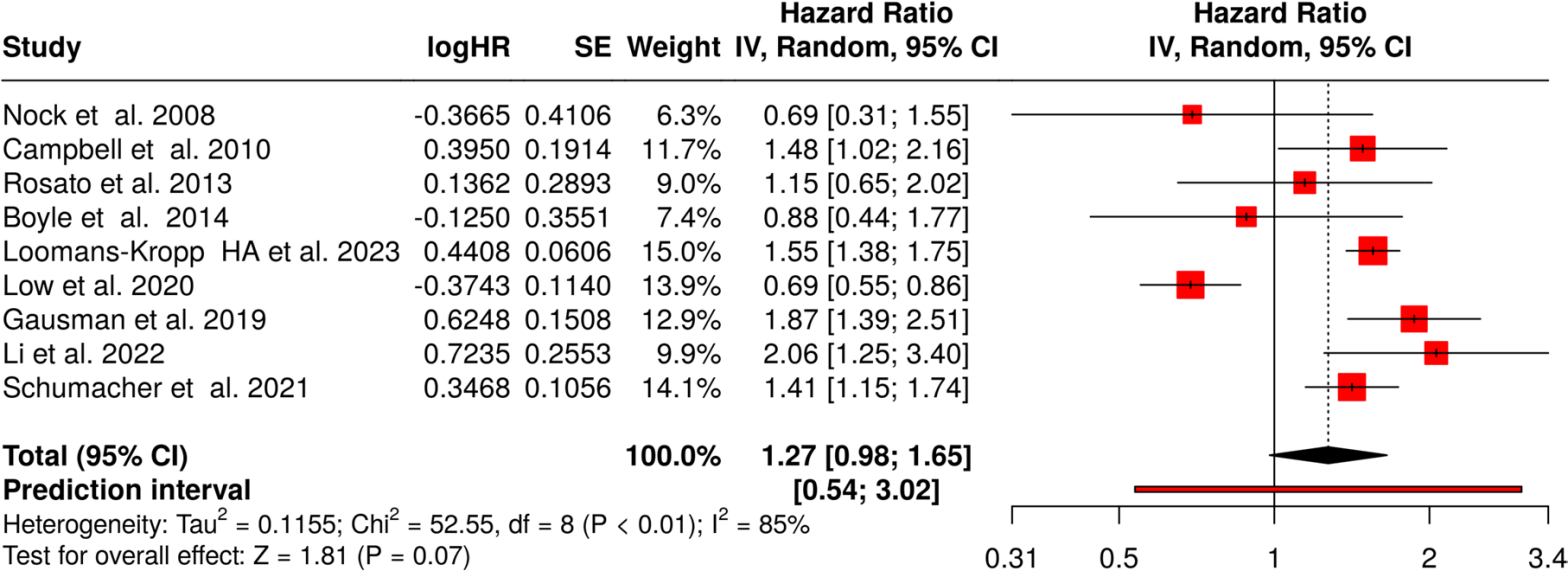
Meta-analysis of case–control studies linking obesity and colorectal cancer in both sexes shows only a marginally significant effect. HR, hazard rate; SE, standard error; CI, confidence interval; IV, inverse variance

A total of nine studies were evaluated [66, 67, 69–75]. Using the random effects model with the inverse variance method to compare hazard rates, no statistically significant difference was observed. The summarized hazard rate (HR) was 1.27 with a 95% confidence interval of 0.98–1.65, and the test for overall effect did not show significance (displayed in Fig. 6).

We have detected a significant heterogeneity, hinting at inconsistent effects in magnitude and/or direction among the studies. The I^2^ value of 84.8% points to the observation that the majority of the observed variability is due to heterogeneity rather than random chance.

The funnel plot does not advocate any potential publication bias. Egger’s test does not provide backing for the presence of a funnel plot asymmetry (intercept: − 1.04, 95% CI − 4.25–2.18, t: − 0.633, *p*-value: 0.547; see Fig. 3D).

**Fig. 7:**
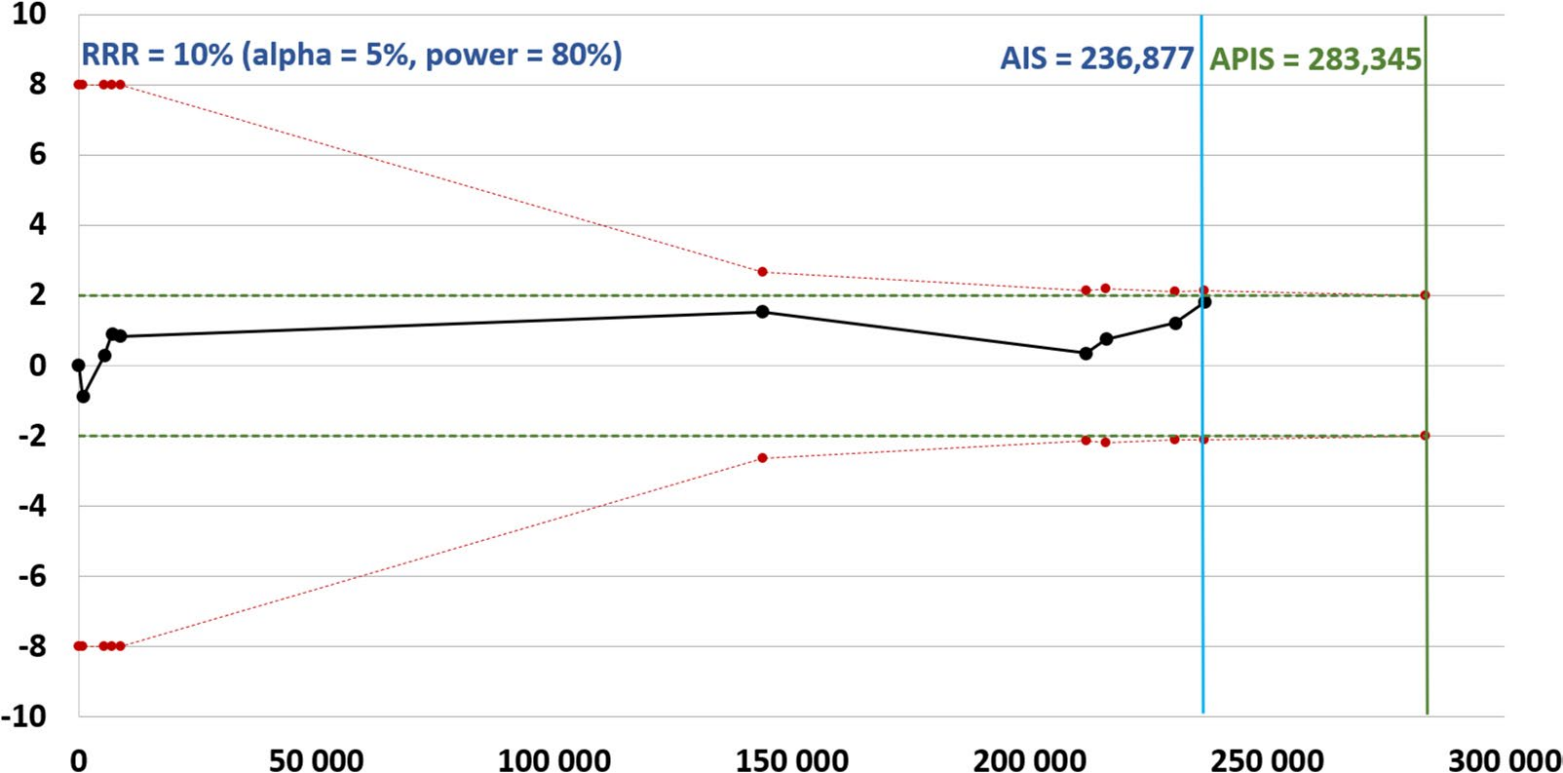
Z-score plot of case–control studies investigating the correlation between obesity and colorectal cancer indicates the need of additional studies to reach a definitive conclusion. AIS, actual information size; APIC, a priori information size; RRR, relative risk reduction

Notably, the total number of cases (*n* = 236,877) included in the case–control analysis is below the a priori information size necessary for reaching statistical significance (*n* = 283,345), suggesting that the number of patients currently included in the case–control analysis is insufficient to draw a decisive conclusion and that further research is needed to validate present findings (Fig. 7).

## Discussion

This meta-analysis provides compelling evidence that overweight and obesity are significant risk factors for CRC. Our results indicate that individuals with overweight or obesity have an elevated risk of CRC, with pooled hazard rates ranging from 1.25 to 1.57, depending on sex.

The findings from 52 cohort studies encompassing 83,251,050 participants show a consistent association between elevated BMI and increased CRC risk. For both men and women, being overweight or obese significantly raises the hazard rate for developing CRC, with men demonstrating a slightly higher risk (HR = 1.57) compared to women (HR = 1.25). These sex-specific differences may be attributable to biological factors, lifestyle differences, and varying fat distribution patterns between men and women. The case–control studies did not show a statistically significant association, which might be due to the smaller sample size compared to the cohort studies.

The increased morbidity and mortality of CRC in overweight and obese individuals can be attributed to several interrelated cellular and molecular mechanisms [104–106]. Overweight and obesity, which are associated with accelerated aging processes and increased biological age [89, 107], appear to similarly elevate the incidence of CRC across various molecular subtypes, as defined by specific molecular markers [108]. One significant factor is chronic inflammation, which is often elevated in individuals with excess body fat [104, 105, 109–112]. Adipose tissue, particularly visceral fat, secretes pro-inflammatory cytokines such as TNF-α, IL-6, and CRP, creating a systemic inflammatory environment [98, 113–121]. An important contributing factor appears to be the increased presence of senescent cells [122]. This chronic inflammation can lead to DNA damage and promote a tumorigenic environment in the colon. Furthermore, it also contributes to the pathogenesis of various other age-related diseases driven by inflammation, such as atherosclerosis [98, 113, 115, 118, 123–131]. Heightened inflammatory status likely also promotes tumor progression and metastasis [106]. Inflammatory mediators, growth factors, and other adipokines secreted from the visceral fat of obese patients likely impact cell proliferation, promote angiogenesis, activate mechanisms involved in invasion and metastasis, contribute to reprogramming energy metabolism, and modulate immune responses [106]. In obese patients, increased adipose tissue leads to higher levels of adipose stromal/stem cells (ASCs) throughout the body, which can influence cancer progression by enhancing tumorigenesis and metastasis through multiple mechanisms, including the recruitment of ASCs to tumors and the production of cytokines and growth factors [132, 133]. Emerging evidence suggests that obesity alters the biological properties of ASCs, further promoting cancer development and spread [132]. Preclinical studies confirm that high fat diet-induced obesity per se significantly increases progression of a mouse colon cancer cell line in an orthotopic transplantation mouse model [134]. Additionally, obesity is associated with insulin resistance and hyperinsulinemia [135]. Elevated levels of insulin and insulin-like growth factor-1 (IGF-1) can promote cellular proliferation and inhibit apoptosis, further contributing to cancer development and progression [106, 136, 137]. Leptin, a hormone produced by adipose tissue, is another key player; in obese individuals, elevated leptin levels can enhance cell proliferation and angiogenesis while inhibiting apoptosis [138–144]. Conversely, adiponectin, which has anti-inflammatory and anti-proliferative effects, is typically reduced in obesity, removing a protective factor against cancer development [145, 146].

Moreover, obesity-induced alterations in the gut microbiota can influence CRC risk [147–149]. Dysbiosis, characterized by an imbalance in the gut microbial community, can lead to the production of carcinogenic compounds and promote an inflammatory state in the colon [147–150].

Furthermore, obesity can induce epigenetic changes that contribute to cancer initiation and/or progression [151–154]. Epigenetic modifications such as DNA methylation, histone modification, and non-coding RNA expression can alter gene expression patterns critical for cell growth, differentiation, and survival [152, 153]. These changes can lead to the activation of oncogenes and the silencing of tumor suppressor genes, facilitating cancer initiation and progression.

Lastly, oxidative stress, prevalent in obese individuals due to increased free fatty acids, adipokines, and dysregulation of proteins involved in production and elimination of ROS, can cause direct DNA damage and promote mutagenesis [155–158].

In addition to obesity-related increases in ROS production, aging itself is associated with a decline in cellular resilience to oxidative stress. A key factor in this decline is the dysfunction of nuclear factor erythroid 2-related factor 2 (Nrf2), a transcription factor that regulates the expression of antioxidant proteins protecting cells against oxidative damage triggered by metabolic stress [159–168]. In younger individuals, Nrf2 effectively maintains cellular redox homeostasis by activating the expression of detoxifying and antioxidant enzymes [86, 88, 160, 169–172]. However, with age, Nrf2 activity diminishes, leading to reduced expression of these protective enzymes and heightened vulnerability to oxidative stress. The dysfunction of Nrf2 in aging cells exacerbates the oxidative damage and inflammation associated with obesity. In an aged organism, the combined impact of obesity-induced oxidative stress and the natural age-related decline in Nrf2 function results in a significantly elevated oxidative burden. This heightened oxidative stress can further accelerate the development and progression of cancer [166]. We posit that in aged obese individuals, the reduced capacity to counteract ROS due to impaired Nrf2 function likely contributes to increased DNA damage, sustained inflammatory responses, and enhanced tumorigenic processes. The cumulative effect of these mechanisms not only increases the risk of CRC development but also contributes to more aggressive tumor phenotypes, leading to higher morbidity and mortality in overweight and obese individuals. Understanding these pathways is crucial for developing targeted therapies and prevention strategies to mitigate CRC risk in this population [166]. In particular, the interplay between obesity-related cellular oxidative stress and age-related decline in oxidative stress resilience underscores the need for targeted interventions that can enhance Nrf2 activity. Strategies aimed at boosting Nrf2 function or mimicking its activity [173] could potentially mitigate the oxidative damage and inflammation driving CRC in obese and aging populations. Thus, understanding the dual impact of obesity and aging on cellular oxidative stress mechanisms is critical for developing effective preventive and therapeutic approaches to reduce CRC risk in these vulnerable groups.

Obesity is not only a significant risk factor for CRC but is also associated with multiple other types of cancer, including cancers of the esophagus, gall bladder, pancreas, breast, endometrium, ovary, thyroid, kidney, and prostate as well as multiple myeloma [8, 174]. This broad association raises the possibility that similar mechanisms, including chronic inflammation and hormonal imbalances, contribute to cancer development across these various organs [8]. Epidemiological studies estimate that 4–38% of cancers at these sites can be attributed to overweight and obesity, depending on the specific cancer type and sex [8]. Further highlighting the impact of obesity on cancer, data from Australia in 2013 indicated that 4.3% of all cancers diagnosed were attributable to overweight and obesity [174]. Analyzing age-specific incidence trends over the past 35 years for obesity-related cancers revealed that the incidence rate ratios (IRRs) for these cancers increased significantly, from 0.77 (95% CI 0.73, 0.81) for those born in 1903 to 2.95 (95% CI 2.58, 3.38) for the 1988 birth cohort, relative to the 1943 cohort [174]. In contrast, IRRs for non-obesity-related cancers remained stable, with non-significant decreases in younger cohorts [174].

Given the significant role of overweight and obesity in CRC [175–182], it is crucial to develop and implement strategies that effectively reduce BMI and to understand their impact on CRC risk. Further research into the effects of such interventions on CRC incidence will provide valuable insights into potential preventive measures, helping to shape more effective public health policies and individual treatment plans.

The potential of pharmacological treatments for obesity and their impact on CRC risk is an emerging area of study that holds promise for both cancer prevention and management. Incretin-based pharmacological interventions aimed at weight loss, such as GLP-1 receptor agonists (e.g., liraglutide and semaglutide), have demonstrated significant efficacy in reducing body weight and improving metabolic profiles in obese individuals [183, 184]. These medications work by enhancing insulin sensitivity, reducing appetite, and promoting satiety, leading to substantial weight loss. The reduction in body weight and improvement in metabolic health associated with these treatments could potentially lower CRC risk by mitigating obesity-related risk factors such as chronic inflammation, insulin resistance, and dyslipidemia. Studies have shown that GLP-1 receptor agonists not only aid in weight loss but also exhibit direct anti-cancer effects. For instance, research has indicated that these drugs can reduce the proliferation of colon cancer cells and induce apoptosis, thereby inhibiting tumor growth [185]. The potential dual benefit of weight reduction and direct anti-cancer activity could make GLP-1 receptor agonists a promising pharmacological option for reducing CRC risk in obese individuals. However, the real-life effect of these medications on CRC risk is not yet fully understood. Clinical trials assessing the efficacy and safety of these weight loss medications, including their direct effects on CRC incidence, are needed to establish their true impact. Much more research is required to determine the long-term outcomes and mechanisms by which these pharmacological treatments might influence CRC risk. This will involve comprehensive studies that include large, diverse populations and long follow-up periods to provide robust evidence on their role in cancer prevention.

Bariatric surgery is another intervention that has demonstrated significant reductions in obesity and associated comorbidities, which may impact incidence of CRC [186, 187]. Post-surgical weight loss leads to improvements in inflammatory markers, insulin sensitivity, and adipokine profiles, potentially contributing to a lower risk of CRC [186–188].

Public health interventions targeting obesity and overweight present a promising area for future research, particularly in understanding how these interventions can causally link to reduced CRC risk [149]. These interventions can range from comprehensive lifestyle programs to specific policy changes and workplace health promotions. Future studies could evaluate the effects of dietary interventions resulting in sustained reduction on BMI, on CRC risk[149]. Research should also investigate the impact of regular physical activity on CRC risk and determine the mediating effect of weight loss. Long-term cohort studies can provide data on how sustained physical exercise, tailored to different age groups and fitness levels, contributes to CRC prevention. Integrating behavioral counseling with dietary and physical activity interventions can be studied to assess its effectiveness in promoting sustained weight loss and reducing CRC risk [149]. The use of digital health tools and mobile applications to support these interventions could also be explored.

Consumption of various obesogenic foods, including sugar-sweetened beverages, has been linked to an increased risk of CRC [189–195]. Policies such as sugar-sweetened beverage taxes [196–199], subsidies for healthy foods, and regulations limiting unhealthy food marketing could be evaluated for their impact on obesity rates and subsequent CRC risk. Comparative studies across different regions implementing varying levels of these policies could provide insights into their effectiveness.

Studies could examine the role of urban planning and the availability of recreational spaces in promoting physical activity and reducing obesity [200]. Longitudinal studies assessing changes in CRC incidence in communities before and after the introduction of such urban planning initiatives would be particularly informative.

Implementing and studying comprehensive workplace health programs that encourage physical activity, healthy eating [149], and regular health screenings can offer insights into reducing obesity [201] and CRC risk. Research could compare CRC incidence in organizations with robust health promotion programs to those without. Investigating the impact of policies that promote work-life balance, such as flexible working hours, on employees’ physical activity levels and dietary habits could be beneficial. This could include assessing the CRC risk reduction in employees who participate in such programs. Investigating the impact of community-based public awareness campaigns on obesity and CRC risk could provide valuable insights. Studies could measure changes in community obesity rates and CRC incidence following targeted educational initiatives. Research could also focus on the long-term effects of school-based nutrition and physical activity programs on childhood obesity and subsequent adult CRC risk. Tracking cohorts of children exposed to these programs into adulthood would help establish the long-term benefits of early intervention. By exploring these diverse intervention strategies through well-designed studies, researchers can identify the most effective approaches to reduce obesity and, consequently, CRC risk. Establishing causal links between public health interventions and reduced CRC incidence will support the development of evidence-based policies and programs aimed at mitigating this significant health risk.

A major strength of this meta-analysis is the comprehensive inclusion of 66 studies, providing a robust assessment of the relationship between overweight, obesity, and CRC risk. The use of both cohort and case–control studies allows for a thorough examination of this association across different study designs and populations. However, the study is not without limitations. Significant heterogeneity among included studies suggests variability in study populations, methods, and potential confounders that could affect the results. Despite rigorous attempts to minimize bias, the potential for residual confounding cannot be entirely excluded. Potential factors contributing to this variability include differences in study populations (e.g., geographic region, age, ethnicity), variations in study design, and differences in how overweight and obesity were defined or measured. Additionally, lifestyle factors such as diet [202], physical activity [203], and access to healthcare likely varied across studies, which could influence CRC risk independently of obesity. Additionally, while funnel plots and Egger’s test did not indicate significant publication bias, the possibility of unreported negative studies cannot be completely ruled out. Given the observed heterogeneity, more research is needed to understand the mechanisms driving sex differences in CRC risk associated with obesity [204]. Potential biological factors contributing to the higher CRC risk in obese men compared to women may include differences in fat distribution, with men more likely to accumulate visceral fat, which is associated with higher inflammation and metabolic dysfunction. Hormonal differences, such as the protective effects of estrogen in women, could also play a role. Additionally, lifestyle factors such as higher rates of smoking and alcohol consumption in men could interact with obesity to increase CRC risk. Additionally, long-term longitudinal studies assessing the impact of weight loss interventions on CRC incidence would offer valuable insights into potential preventive measures [149, 205]. Future research should explore the role of regular health screenings and weight management counseling in primary care settings. Studies could evaluate the effectiveness of integrating weight management into routine CRC screening and prevention programs, assessing how early intervention impacts long-term CRC risk [149].

In conclusion, this meta-analysis highlights the significant association between overweight, obesity, and increased CRC risk. With the obesity epidemic rising in regions like the United States and the European Union, there is an urgent need for effective public health interventions to address this modifiable risk factor. By mitigating obesity, it may be possible to substantially reduce the burden of CRC and improve population health outcomes. Public health policymakers and healthcare providers should prioritize obesity prevention and treatment as a key strategy in cancer prevention efforts. Enhanced public awareness, lifestyle modifications, and targeted interventions could play a critical role in reducing the incidence of CRC and other diseases [107, 206, 207] linked to overweight and obesity, ultimately leading to better health outcomes and reduced healthcare costs.
